# Immune checkpoint inhibition improves antimyeloma activity of bortezomib and STING agonist combination in Vk*MYC preclinical model

**DOI:** 10.1007/s10238-022-00878-1

**Published:** 2022-08-31

**Authors:** Olga Sokolowska, Anna Rodziewicz-Lurzynska, Zofia Pilch, Hanna Kedzierska, Justyna Chlebowska-Tuz, Anna Sosnowska, Anna Szumera-Cieckiewicz, Kamil Sokol, Joanna Barankiewicz, Aleksander Salomon-Perzynski, Olga Ciepiela, Ewa Lech-Maranda, Jakub Golab, Dominika Nowis

**Affiliations:** 1 Laboratory of Experimental Medicine, Centre of New Technologies, University of Warsaw, Banacha, 2C, 02-097 Warsaw, Poland; 2 Central Laboratory, University Clinical Center of Medical University of Warsaw, Banacha 1A, 02-097 Warsaw, Poland; 3 Department of Immunology, Medical University of Warsaw, Nielubowicza 5, 02-097 Warsaw, Poland; 4 Department of Pathology, Maria Sklodowska-Curie National Research Institute of Oncology, Roentgena 5, 02-781 Warsaw, Poland; 5 Diagnostic Hematology Department, Institute of Hematology and Transfusion Medicine, Indiri Ghandi 14, 02-776 Warsaw, Poland; 6 Department of Hematology, Institute of Hematology and Transfusion Medicine, Indiri Ghandi 14, 02-776 Warsaw, Poland; 7 Department of Laboratory Medicine, Medical University of Warsaw, Banacha 1A, 02-097 Warsaw, Poland; 8 Centre of Preclinical Research, Medical University of Warsaw, Banacha 1B, 02-097 Warsaw, Poland; 9 Laboratory of Experimental Medicine, Medical University of Warsaw, Nielubowicza 5, 02-097 Warsaw, Poland

**Keywords:** Multiple myeloma, Bortezomib, STING, cGAMP, PD-1, Immune checkpoint inhibitor

## Abstract

Multiple myeloma (MM), a hematological malignancy of plasma cells, has remained incurable despite the development of novel therapies that improve patients’ outcome. Recent evidence indicates that the stimulator of interferon genes (STING) pathway may represent a novel target for induction of antitumor immune response in multiple myeloma. Here, we investigated antitumor effects of STING agonist with bortezomib with or without checkpoint inhibitor in the treatment of MM. Methods: STING expression in bone marrow plasma cells of 58 MM patients was examined by immunohistochemical staining. The effectiveness of the proposed therapy was evaluated in vivo in a syngeneic transplantable mouse model of MM (Vĸ*MYC) in immunocompetent mice. Flow cytometry was used to assess tumor burden and investigate activation of immune response against MM. ELISA was performed to measure serum inflammatory cytokines concentrations upon treatment. Results: Combining a STING agonist [2′3′-cGAM(PS)_2_] with bortezomib significantly decreased tumor burden and improved the survival of treated mice compared to either of the compounds used alone. The combination treatment led to secretion of pro-inflammatory cytokines and increased the percentage of neutrophils, activated dendritic cells and *T* cells in the tumor microenvironment. However, it resulted also in increased expression of PD-L1 on the surface of the immune cells. Addition of anti-PD1 antibody further potentiated the therapeutic effects. Conclusions: Our findings indicate high antimyeloma efficacy of the three-drug regimen comprising bortezomib, STING agonist, and a checkpoint inhibitor.

**Supplementary Information:**

The online version contains supplementary material available at 10.1007/s10238-022-00878-1.

## Background

Multiple myeloma (MM) is a malignancy characterized by the clonal proliferation of plasma cells in the bone marrow. It is the second most common hematological malignancy worldwide [1]. Despite significant progress in treatment over the last decades, MM remains an incurable disease due to recurrent relapses. Complex genomic landscape, clonal heterogeneity, progressive immune dysfunction and immunosuppressive microenvironment are some of the reasons that make MM so difficult to cure [2, 3]. With the approval of monoclonal antibodies against CD38 (daratumumab and isatuximab) and SLAMF7 (elotuzumab), as well as anti-BCMA chimeric antigen receptor T cells (CAR-T, idecabtagene vicleucel, ciltacabtagene autoleucel), immunotherapy has emerged as a promising treatment option for MM patients. Various immunotherapeutic approaches are now under preclinical and clinical development. These include monoclonal antibodies, CAR-T cells, bispecific T-cell engagers, bispecific antibodies or antibody–drug conjugates [4].

STING is a transmembrane protein, which functions as an adaptor protein in the innate immune pathway triggered by the detection of cytosolic nucleic acid ligands. One of the most important DNA sensor is cyclic GMP-AMP synthase (cGAS) which secretes natural ligands of STING known as cGAMPs [5]. Binding of cyclic dinucleotides to STING activates downstream signaling that involves tank-binding kinase 1 (TBK1), interferon regulatory factor 3 (IRF3), nuclear factor kappa B (NF-kB) and leads to the production of type I interferons (IFNs), tumor necrosis factor *α* (TNF*α*) as well as other inflammatory cytokines and chemokines [6]. It is well established that type I IFNs produced upon activation of the STING pathway are essential for CD8^+^ T cell cross-priming by tumor antigens and induction of adaptive anticancer immune response [7]. Numerous studies identified STING as a promising target for cancer immunotherapy [7–11] and modified synthetic STING agonists are currently tested in clinical setting, mostly in combination with anti-programmed death 1 (PD-1) antibodies [12].

Recent evidence suggests that activation of STING-mediated signaling can synergize with proteasome inhibitor bortezomib in the treatment of MM [13]. Here, we investigate the antimyeloma potential of two-drug combination comprising bortezomib and STING agonist in transplantable Vĸ*MYC model, a well-established syngeneic murine immunocompetent model for preclinical studies of drug efficacy in MM [14]. We show that the proposed combination treatment results in the improved survival and development of anticancer immune response in vivo and that adding PD-1 blocking antibody can further increase survival advantage.

## Methods

### Reagents

cGAM(PS)_2_ was purchased from Invivogen, bortezomib was purchased from Adamed. *InVivo*MAb anti-PD-1 monoclonal antibody (RMP1-14, #BE0146) and matched isotype control (2A3, #BE0089) were purchased from Bio X Cell.

### Immunoblotting

Cell lysis was performed in RIPA buffer (Sigma-Aldrich) supplemented with protease and phosphatase inhibitor cocktail (Roche). Samples were resolved by SDS-PAGE and transferred onto nitrocellulose membranes (GE Healthcare). Antibodies were obtained from Cell Signaling Technology: STING (#13647), anti-rabbit HRP conjugated (#7074), and Sigma-Aldrich: β-actin (#A3854). The blots were visualized with Clarity and Clarity Max substrates (Bio-Rad) using Amersham Imager 680 (GE Healthcare).

### Immunohistochemistry

A retrospective group of 58 patients diagnosed according to the clinical and histopathological criteria 2017 World Health Organization classification with multiple myeloma were enrolled for the study. All cases of plasma cell myeloma were histopathologically diagnosed according to WHO 2017 classification criteria and IMWG: International Myeloma Working Group; the immunohistochemistry panel includes CD138, CD56, CD19, CD20, or BSAP/PAX5 (in selected cases), kappa, and lambda; the percentage of plasma cell myeloma infiltration is always provided. Immunohistochemistry on formalin-fixed paraffin-embedded sections was performed using an automated stainer (Dako) and anti-TMEM173 (STING) antibody (Abcam, #189430), rabbit polyclonal, 1:200, pH 6.0. EnVision Detection System (Dako) was used for signal detection. Positive control included the tonsil epithelium; the internal control was strong reaction in erythroid precursors (erythroblasts). Negative (isotype) control stainings were performed using a ready to use Universal Negative Control for IS-Series Rabbit Primary Antibodies (code nr IS600; Dako) and FLEX Negative Mouse Control (cocktail of mouse IgG1, IgG2a, IgG2b, IgG3 and IgM; code nr IR750; Dako). Samples were considered negative (no reaction), weakly (weak staining or strong reaction in < 30% cells) or strongly (strong reaction in ≥ 30% cells) positive for STING. All stained sections were independently reviewed for expression of STING by 2 pathologists (ASC and KS). The microphotographs were taken by a microscope DP72 Olympus BX63 camera (Olympus).

### In vivo* studies*

All animal experiments were approved by the Local Ethics Committees (at the Medical University of Warsaw, approval No 86/2015 and II Local Ethics Committee in Warsaw, approval No WAW2/055/2020) and carried out in accordance with the requirements of EU (Directive 2010/63/EU) and Polish (Dz. U. poz. 266/15.01.2015) legislation. C57BL/6 WT mice were purchased from the Mossakowski Medical Research Centre Polish Academy of Sciences, MPYS^−/−^/Tmem173tm1Camb (STING KO) mice were provided by Prof. Vincenzo Cerundolo, (MRC Human Immunology Unit, Weatherall Institute of Molecular Medicine, UK).

### Tumor transplantation and therapy

C57BL/6 wild-type or STING KO mice were transplanted with 0.75 × 10^6^ Vĸ*MYC cells (a kind gift from Prof. Leif Bergsagel, Mayo Clinic College of Medicine, USA) [14, 15] via the tail vein. Three weeks post-transplantation, the development of MM was confirmed by detection of monoclonal immunoglobulins (M-spike) with serum protein electrophoresis (SPEP)(HYDRASYS, Sebia Hydragel). Bortezomib (0.6 mg/kg) was injected intraperitoneally (IP) on days 1, 4, 7, 10, 2′3′-cGAM(PS)_2_ (50 μg) was given IP on days 2, 5, 8, anti-PD-1 (10 mg/kg) was injected IP on days 2, 5, 8, 11, 14 after beginning of the treatment. M-spikes were assessed weekly as a marker of tumor response and gamma globulin/albumin ratio post-treatment for each individual M-spike was normalized by the gamma globulin/albumin.

### Detection of MM in the spleen

Three weeks after the beginning of the treatment the spleens were harvested, mashed through a 70 µm nylon mesh (Corning) into single-cell suspension and the red blood cells were lysed using ACK solution (Thermo Fisher Scientific). Cells were stained with live/dead stain (anti-B220 eFluor 450 (eBioscience) and anti-CD138 PE (Becton Dickinson) antibodies, and analyzed by flow cytometry on LSR II Fortessa (Becton Dickinson) instrument. MM cells gating strategy is presented in Supplementary Fig. 1.

### Multiplex cytokine profiling

Serum samples collected 6 h after the first and third doses of cGAM(PS)_2_ were subjected to multiplexed cytokine analysis using BD Cytometric Bead Array Mouse Inflammation Kit (Becton Dickinson), according to the manufacturer’s protocol. Flow cytometry was performed on a LSR II Fortessa (Becton Dickinson). Data analysis was performed with FACSDiva 8.0 and FCAP Array software (Becton Dickinson).

### ELISA

Serum was obtained 4 h after the first dose of STING agonist. ELISA was performed for IFN-*β* using LumiKine Xpress mIFN-*β* 2.0 (InvivoGen) according to the manufacturer’s instructions.

### Detection of immune cells in tumor microenvironment

One day and 1 week after treatment, the spleens were harvested, mashed through a 70-µm nylon mesh (Corning) into single-cell suspension and the red blood cells were lysed using ACK solution (Thermo Fisher Scientific). Cells were stained with the Aqua or NIR Zombie Fixable Viability Kit (BioLegend) according to the manufacturer’s instructions. Then, cells were stained with panel 1 or panel 2 antibodies (Supplementary Tables 1, 2) and analyzed by flow cytometry on a LSR II Fortessa (Becton Dickinson). For multicolor staining panels setup appropriate isotype and FMO (fluorescence minus one) controls were included. Only live, single-cell events were analyzed. Gating strategies to identify neutrophils, dendritic cells, *T* cells and macrophages are presented in the Supplementary Figs. 2, 3, 4, 5, respectively.

### Statistical analysis

Statistical analyses were carried out with GraphPad Prism 7 software. Statistical tests included one-way ANOVA followed by Dunnett’s post hoc test or two-way ANOVA followed by Bonferroni’s post hoc test. Survival data were evaluated with the Kaplan–Meier analysis, and comparison of survival curves was made with the log-rank test. *P*-value < 0.05 was considered significant: *p < 0.05, **p < 0.01, ***p < 0.001. Analysis of relative expression was performed with LightCycler 480 Software (LCS480 1.5.1.62). For statistical analysis of clinical parameters of MM patients, STING samples were divided into two groups: STING-negative (with no IHC reaction) and STING-positive (weak or strong IHC reaction). Categorical variables were compared using Chi-squared or Fisher test depending on the number of observations in each 2-by-2 table. Continues variables were compared using the t-Student test if they followed normal distribution or Wilcoxon test if they did not follow normal distribution. The distribution of the variables was checked by plotting histograms. Survival function with 95% confidence intervals was estimated using the Kaplan–Meyer method. To estimate hazard ratios and 95% confidence intervals, the proportional hazard Cox model was used. All tests were two-sided and were performed at a 0.05 significance level. All analyses were performed using software: Statistica v. 13.1 and MedCalc v. 20.027.

## Results

### STING expression is frequently downregulated in multiple myeloma cells

**Fig. 1:**
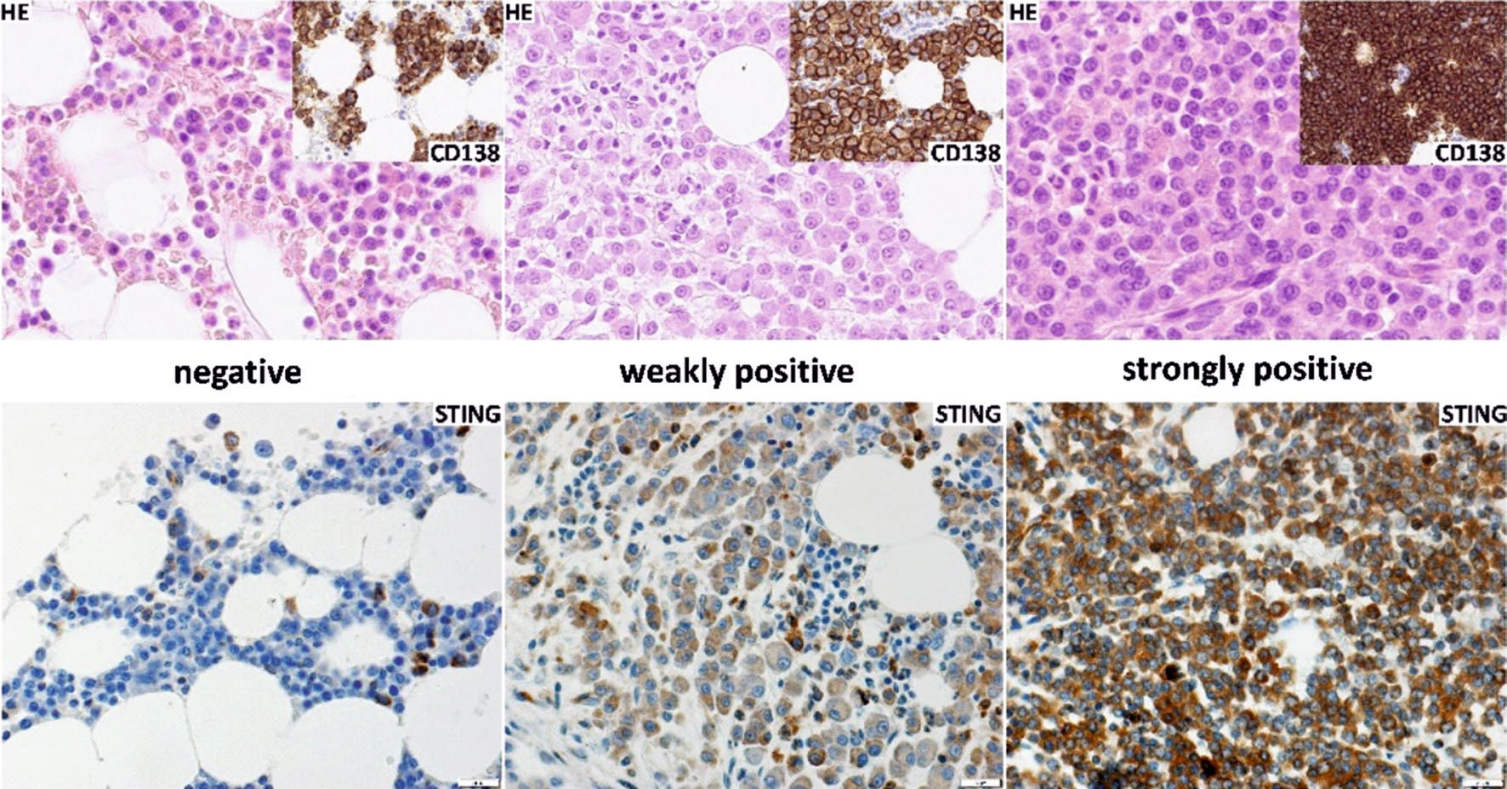
STING is expressed in human MM cells. Lower panel: STING expression in MM cells evaluated by IHC; exemplary negative (left), weakly positive (middle) and strongly positive (right) samples; upper panel: H&E and anti-CD138 staining of the corresponding samples. Bar indicates 20 μm

STING signaling has been reported to be largely impaired in a number of cancer types including melanoma, ovarian and colorectal cancer [16–18]. To assess whether STING expression is deregulated in MM cells we performed immunohistochemical examination of bone marrow trepanobiopsy specimens from newly diagnosed MM patients. The results showed positive STING staining in MM cells in 47% of cases, of which 31% exhibited strong staining (Fig. 1, Table 1). Thus, over half of the patient-derived samples lacked STING in the MM cells. Due to small group sizes, no correlations were detected between the intensity of STING staining, clinical parameters of MM patients’, response rate to the first-line treatment or probability of survival (Supplementary Table. 3, Supplementary Fig. 6).

**Table 1:** Intensity of immunohistochemical staining with antibody against STING in a panel of bone marrow samples from MM patients

Negative	Weakly positive	Strongly positive
53% (31/58)	16% (9/58)	31% (18/58)

### *Activation of STING pathway potentiates antimyeloma effects of bortezomib *in vivo

**Fig. 2:**
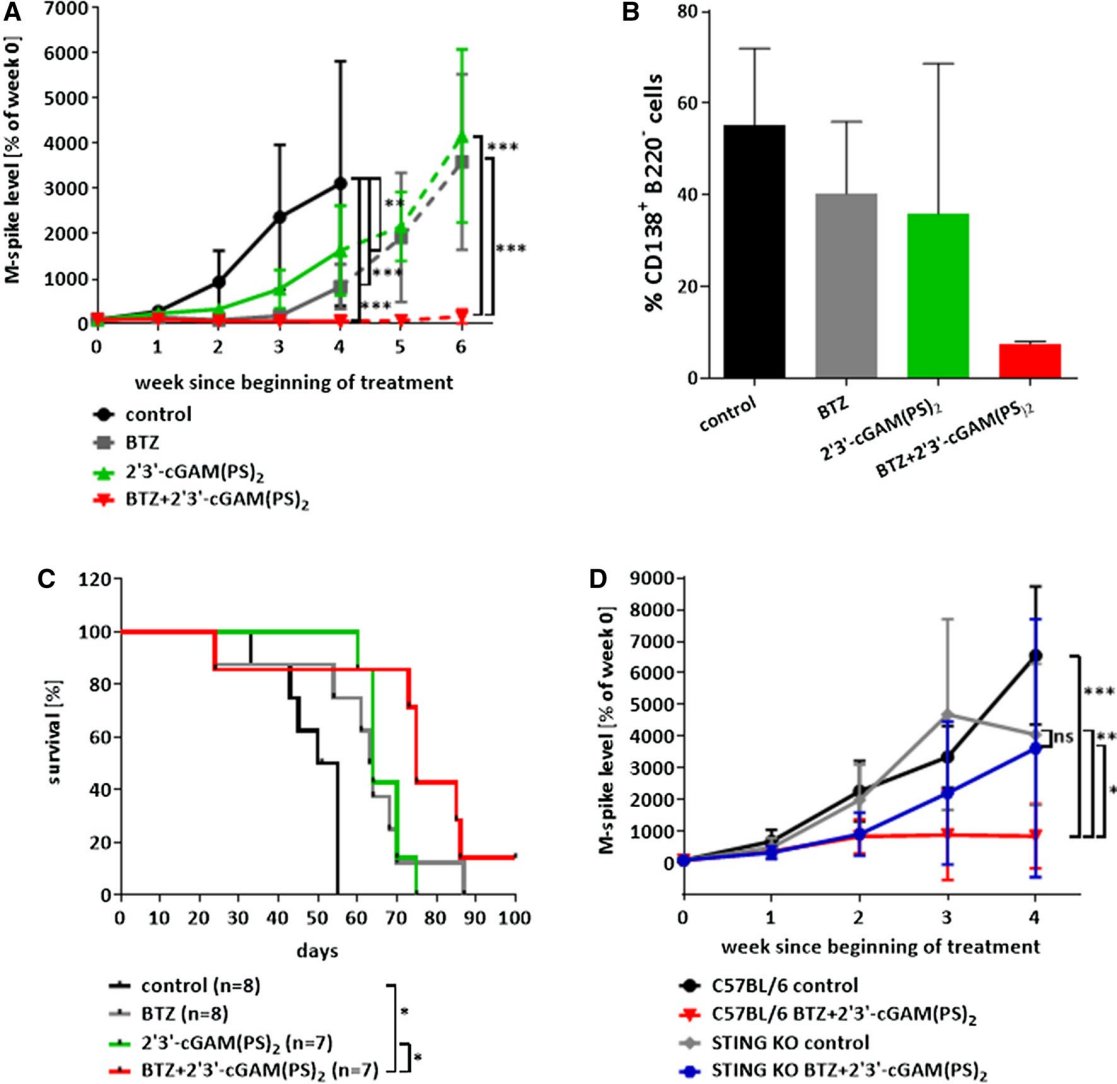
STING agonist augments bortezomib-mediated antitumor effects in vivo. C57BL/6 mice (**A-C**) or STING KO mice (**D**) were transplanted with 0.75 × 10^6^ Vĸ*MYC cells and 3 weeks later treated with intraperitoneal administration of PBS (control), bortezomib (BTZ, 0.6 mg/kg on days 1, 4, 7, 10), 2′3′-cGAM(PS)_2_ (50 μg on days 2, 5, 8), or combination of BTZ and 2′3′-cGAM(PS)_2_. **A** M-spike changes in C57BL/6 mice since the beginning of the treatment. Graph depicts the mean ± SD. Weeks 0–4 and 4–6 were analyzed separately due to different number of experimental groups. Experimental groups consisted of 7–8 mice. **p < 0.01, ***p < 0.001; two-way ANOVA followed by Bonferroni’s multiple comparisons test. **B** Percentage of MM cells (CD138^+^B220^−^) in the spleens 3 weeks after beginning of the treatment analyzed by flow cytometry. No significant differences were shown by statistical analysis; *n* = 5; one-way ANOVA followed by Tukey’s post hoc test. **C** Kaplan–Meier survival curves of mice. *p < 0.05, log-rank test, *n* = 7–8. **D** M-spike changes in time in STING KO mice bearing Vĸ*MYC cells. Graph depicts the mean ± SD. Experimental groups consisted of 5–8 animals, *p < 0.05, **p < 0.01, ***p < 0.001; two-way ANOVA followed by Bonferroni’s multiple comparisons test

As the IHC data showed (Table 1), the majority of MM cells do not express STING. In order to evaluate whether pharmacologic activation of STING could augment antimyeloma activity of bortezomib in vivo, we chose syngeneic Vκ*MYC immunocompetent MM murine model [14], that lacks STING expression (Supplementary Fig. 7). C57BL/6 mice were transplanted with 0.75 × 10^6^ Vκ*MYC cells and after 3 weeks bortezomib and/or 2′3′-cGAM(PS)_2_ (a chemically modified cGAMP with enhanced half-life in the blood [19]) treatment was initiated and continued for 10 days. The disease progression was monitored with changes in M-spike values, as a validated indicative of MM tumor burden [14]. The data from weeks 1 to 4 and 5 to 6 were analyzed separately, because most animals in the control group died by week 5. At week 4 M-spike values in all treated groups were significantly reduced in comparison with the controls (Fig. 2A). At week 6, the combination treatment resulted in the highest reduction of M-spike values in comparison with single agent-treated mice (Fig. 2A). Moreover, at week 3 post-treatment initiation the percentage of MM cells in the spleen of combination-treated mice was lower than in all other groups (Fig. 2B), complementing the M-spike data (Fig. 2A). The combination treatment prolonged survival of Vκ*MYC-bearing mice compared to controls and STING agonist only treated group (Fig. 2C). The antimyeloma effects of the combination treatment in the Vκ*MYC model are STING-dependent, as they are significantly abrogated in STING KO mice bearing these cells (Fig. 2D, Supplementary Fig. 8). Taken together, our data implicate that STING agonist improves antimyeloma activity of bortezomib in vivo.

### Combination therapy leads to the activation of the immune system

**Fig. 3:**
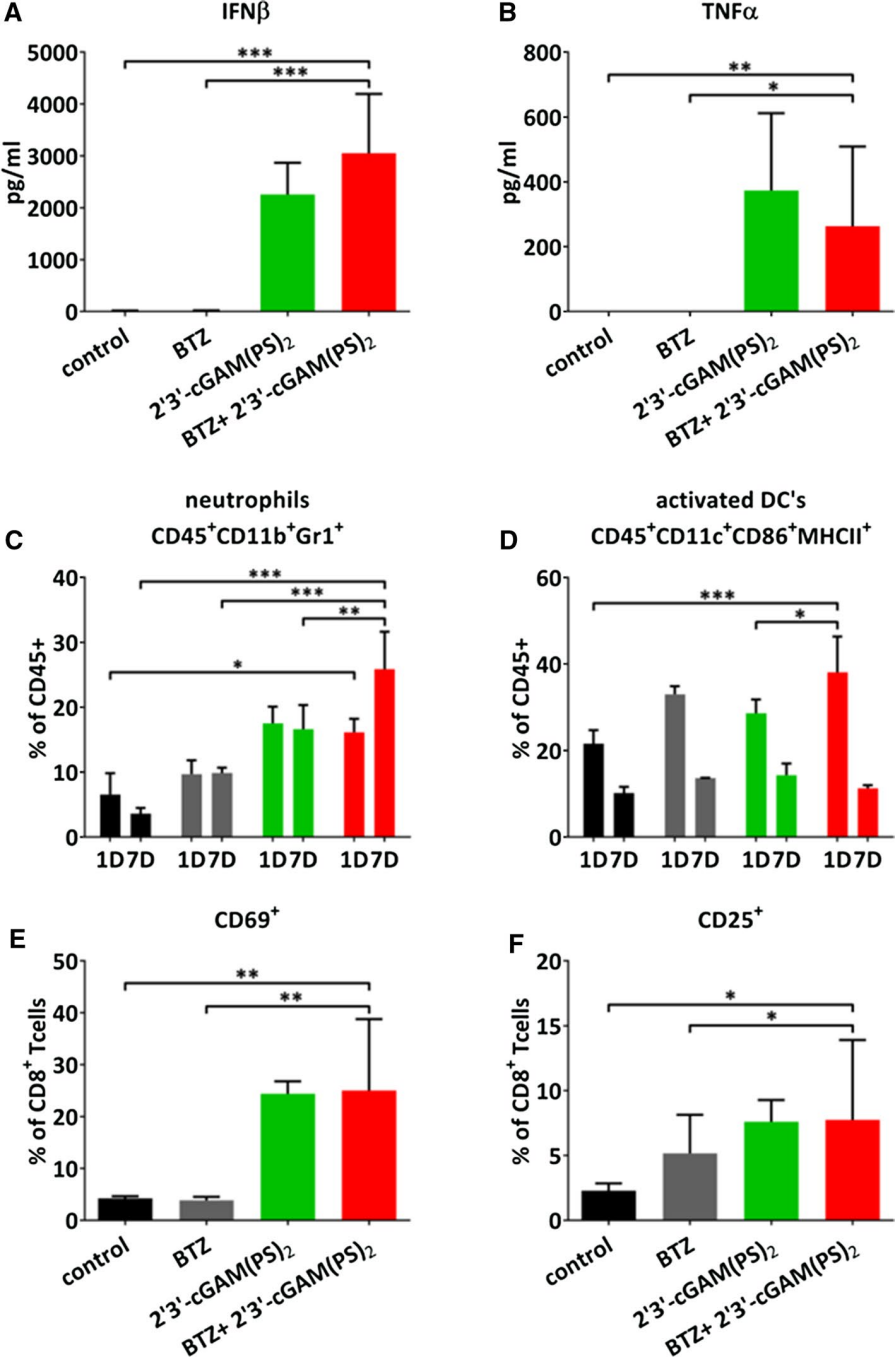
STING agonist and bortezomib combination induces antitumor immunity. C57BL/6 mice were transplanted with 0.75 × 10^6^ Vĸ*MYC cells and treated like in Fig. 2. Experimental groups consisted of 3 mice. **A** IFNβ serum concentration 4 h after first administration of 2′3′cGAM(PS)_2_ was analyzed by ELISA. Data show means ± SD. ***p < 0.001; one-way ANOVA followed by Dunnett’s post hoc test versus combination treated group. **B** TNF*α* concentration in serum samples collected 6 h after first administration of 2′3′cGAM(PS)_2_ was quantified by flow cytometry. Data show means ± SD. *p < 0.05, **p < 0.01; one-way ANOVA followed by Dunnett’s post hoc test versus combination treated group. **C-F** Mouse spleens were harvested 1- or 7-days post-treatment. Cells were immuno-phenotyped for neutrophils (**C**), activated dendritic cells (DC’s) (**D**), CD69^+^CD8^+^ T cells (**E**), and CD25^+^CD8^+^ T cells (**F**). Graph depicts the mean ± SD. *p < 0.05, **p < 0.01, ***p < 0.001; two-way ANOVA followed by Bonferroni’s multiple comparisons test versus combination treated group

Next, we aimed to explore potential mechanisms underlying the in vivo antimyeloma activity of therapy combining bortezomib and a STING agonist. To this end, we measured serum inflammatory cytokines concentrations in the Vκ*MYC-bearing immunocompetent mice after first and last doses of 2′3′-cGAM(PS)_2_ using ELISA and cytometric bead-based multiplex assay. Both STING agonist alone and in combination with bortezomib increased concentrations of IFN*β* and TNF*α* (Fig. 3A-B), as well as MCP-1 (Supplementary Fig. 9A) in treated mice. STING agonist alone, but not in combination with bortezomib, also induced IL-6, IL-10 and IFN*γ* (Supplementary Fig. 9B-D, respectively). To further elucidate the immune mechanisms responsible for the therapeutic effects of the STING agonist and bortezomib combination, we evaluated immune cell infiltration within the tumor microenvironment with flow cytometry. In the spleens of the combination-treated mice on day 1 post-therapy, we observed a statistically significant increase in the percentage of neutrophils as compared with control (Fig. 3C). On day 7 increase in the percentage of neutrophils in the combination-treated mice was statistically significant as compared with all other experimental groups (Fig. 3C). Moreover, combination treatment led to accumulation of activated dendritic cells in the spleens on day 1 as compared to controls and STING agonist-treated group (Fig. 3D) but did not change the percentage of macrophages (Supplementary Fig. 9E). We have also observed an increased percentage of activated CD69^+^ CD8^+^ *T* cells (Fig. 3E) as well as CD25^+^ CD8^+^ *T* cells (Fig. 3F) in the spleens of the combination-treated mice on day 1 post-therapy. All treatment regimens downregulated PD-1 on CD8^+^ *T* cells present in the MM spleen (Supplementary Fig. 9F). Collectively, our results indicate that combination of bortezomib with STING agonist activates immune response against MM.

### PD-1 blockade improves the therapeutic potential of bortezomib and STING agonist combination

**Fig. 4:**
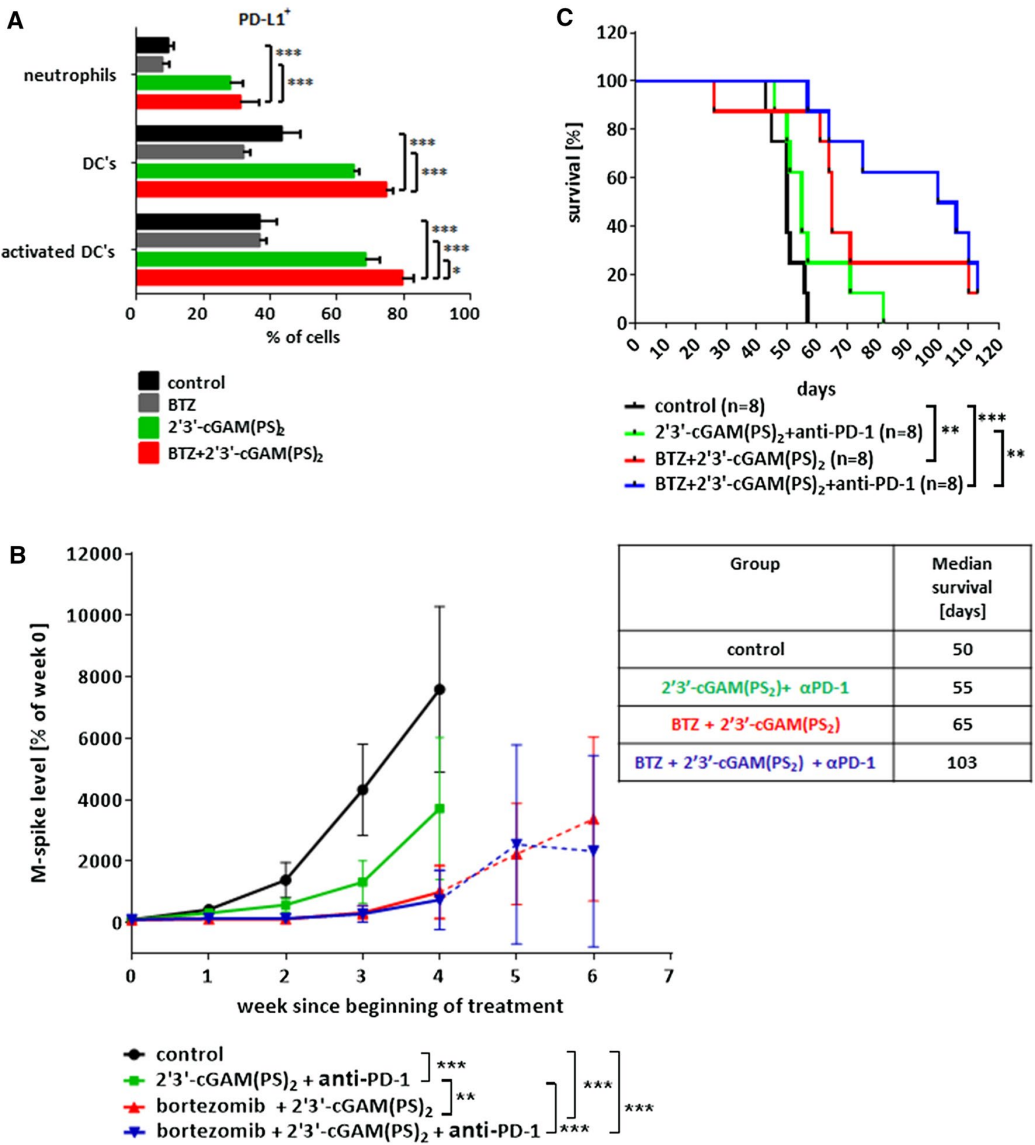
Addition of checkpoint inhibitor to STING agonist and bortezomib combination results in survival advantage. C57BL/6 mice were transplanted with 0.75 × 10^6^ Vĸ*MYC cells and treated like in Fig. 2. **A** PD-L1 expression on the surface of immune cells in the spleen 1 day post-treatment analyzed by flow cytometry. Graph depicts the mean ± SD. *p < 0.05, ***p < 0.001; two-way ANOVA followed by Bonferroni’s multiple comparisons test versus combination treated group. Experimental groups consisted of 3 mice. **B**, **C** C57BL/6 mice were transplanted with 0.75 × 10^6^ Vĸ*MYC cells and 3 weeks later treated with intraperitoneal administration of PBS (control), 2′3′-cGAM(PS)_2_ and anti-PD-1 monoclonal antibody (*α*PD-1), bortezomib (BTZ) and 2′3′-cGAM(PS)_2_, or triple combination. BTZ (0.6 mg/kg) was administered on days 1, 4, 7, 10; 2′3′-cGAM(PS)_2_ (50 μg) on days 2, 5, 8; *α*PD-1 (10 mg/kg) on days 2, 5, 8, 11, 14 after beginning of the treatment. **B** M-spike changes since the beginning of the treatment. Graph depicts the mean ± SD. **p < 0.01, ***p < 0.001; two-way ANOVA followed by Bonferroni’s multiple comparisons test. **C** Kaplan–Meier survival curves of mice. **p < 0.01, ***p < 0.001, log-rank test. Median animal survival in the experimental groups [in days]

Activation of STING-mediated signaling is known to induce expression of programmed death-ligand 1 (PD-L1) [8]. We found that bortezomib and 2′3′-cGAM(PS)_2_ combination therapy leads to increased expression of PD-L1 on the surface of neutrophils and dendritic cells in the spleens of Vκ*MYC-bearing mice (Fig. 4A). Since PD-L1/PD-1 interactions could potentially restrain anticancer immunity induced by STING activation, we evaluated if adding an immune checkpoint inhibitor blocking PD-1 function to the bortezomib and STING agonist treatment could enhance the anti-MM efficacy in the Vκ*MYC-bearing mice. Mice were treated with either 2 or 3 agents: bortezomib, 2′3′-cGAM(PS)_2_, and anti-PD-1 monoclonal antibody (*α*PD-1). Although combining STING agonist with *α*PD-1 showed only moderate anticancer efficacy as measured by serum M-spike levels, mice treated with a combination of bortezomib, STING agonist and/or *α*PD-1 showed the highest reduction of the monoclonal protein (Fig. 4B). Importantly, addition of *α*PD-1 prolonged survival of STING agonist and bortezomib-treated mice (Fig. 4C).

## Discussion

Immunotherapeutic approaches for MM brought mostly unsatisfactory therapeutic outcomes. Over the recent years, this trend has started to change. Several novel immune-based therapies are validated in combination with current standard-of-care drugs. Here, we provide the rationale for a novel therapeutic regimen for patients with MM that combines bortezomib with STING agonist and an immune checkpoint inhibitor—an anti-PD-1 antibody.

Recently, Gulla et al. showed reduction of tumor growth after treatment with bortezomib and intratumoral administration of synthetic cyclic dinucleotide STING agonist ADU-S100 in 5TGM1 xenograft model in C57BL/KaLwRij mice [13]. However, 5TGM1 cells are sensitive to direct killing by STING agonist treatment in vitro [13]. Our results show that STING expression is recurrently suppressed in MM cells in patient-derived samples, which underlines importance of non-direct antitumor activity of STING agonists in the treatment of MM. To recapitulate more clinically relevant conditions, we used Vĸ*MYC model, characterized by the absence of STING protein in MM cells. Vĸ*MYC is a well-established syngeneic murine immunocompetent model that was shown to be an effective preclinical tool to test new MM therapies [14]. We demonstrated that combination treatment with IP administered bortezomib and STING agonist results in reduced tumor burden and significant survival advantage. Accumulating evidence suggests that the antitumor effect of STING pathway activation relies mostly upon secretion of type I interferons, which facilitates activation of dendritic cells and cross-priming of tumor-specific CD8^+^ T cells [20, 21]. We observed that combination treatment exerts immunomodulatory effects, which include significantly enhanced pro-inflammatory cytokine release and increased percentage of activated dendritic cells within the tumor microenvironment. Interestingly, upon combination treatment we observed most prominent accumulation of neutrophils within the tumor microenvironment. This finding is consistent with a previous report showing enhanced recruitment of neutrophils in a MMTV-PyMT murine transgenic breast tumor model after murine STING agonist DMXAA treatment. In this study, neutrophils were necessary for an optimal recruitment and activation of other immune cell populations and inhibition of tumor growth [22]. Moreover, we observed that CD8^+^ T cells from Vĸ*MYC-bearing mice express high levels of exhaustion marker PD-1, which has also been noticed in patients with MM [23]. We showed that combination treatment reduced the co-expression of PD-1 and increased co-expression of activation markers CD69 and CD25.

In addition, we observed that two-drug combination comprising bortezomib and STING agonist induce expression of PD-L1 on the surface of the immune cells in myeloma microenvironment. We showed that addition of *α*PD-1 further prolonged survival of MM-bearing mice. Although in the syngenetic tumor models in C57BL/6 mice PD-1 has been shown to be expressed mainly on CD8^+^ T cells [24], it might be also present on different types of both innate and adaptive immune cells, such as NK cells, NKT cells, monocytes, macrophages, B cells as well as CD4^+^ T cells including Tregs [25]. Thus, we cannot exclude the possibility that the efficacy of anti-PD-1 antibody in Vk*MYC-bearing mice might also be dependent on the modulation of function of not only CD8^+^ T cells, but also other immune cells populations. While US Food and Drug Administration terminated myeloma anti-PD-1 plus lenalidomide/pomalidomide and dexamethasone clinical trials due to an increased risk of death in the pembrolizumab arms [26, 27], no specific adverse events were attributed to checkpoint inhibition, suggesting that other combination treatments with PD-1 blocking antibody are possible for MM. Some new combination therapies have already shown survival advantages in preclinical myeloma models [28–30]. Our study identifies bortezomib, STING agonist and *α*PD-1 treatment as potent antimyeloma therapy, characterized by the longest median survival time.

## Conclusions

In summary, our study validates STING as a therapeutic target in MM and proposes novel combination treatments with bortezomib, STING agonist and an immune checkpoint inhibitor, with augmented immunotherapeutic effects and promising antitumor efficacy in vivo.

## Supplementary Information

Below is the link to the electronic supplementary material.

Supplementary file1 (DOCX 1720 KB)

## Data Availability

All data generated or analyzed during this study are included in this published article [and its supplementary information files].
