# Supplementary material for: Immune checkpoint inhibition improves antimyeloma activity of bortezomib and STING agonist combination in Vk*MYC preclinical model
Source: Clin Exp Med. 2022 Aug 31;23(5):1563–72. doi: 10.1007/s10238-022-00878-1 (PMC10460740; doi:10.1007/s10238-022-00878-1)
Supplement: Supplementary file 1 — Supplementary file1 (DOCX 1720 KB) [file 10238_2022_878_MOESM1_ESM.docx]

Supplementary Table 1. List of antibodies used in the staining – panel 1.

| **Antibody target** | **Clone** | **Source** | **Catalog no.** | **Dilution** | **Fluorochrome** |
| --- | --- | --- | --- | --- | --- |
| CD45.2 | 104 | eBioscience | #25-0454-82 | 1:200 | PE-Cy7 |
| CD11b | M1/70 | Becton Dickinson | #563015 | 1:100 | BV 605 |
| F4/80 | BM8 | Invitrogen | #MF48028 | 1:200 | Pacific blue |
| Gr1 | RB6-8C5 | BioLegend | #108406 | 1:200 | FITC |
| CD11c | N418 | eBioscience | #12-0114-82 | 1:200 | PE |
| CD86 | GL1 | eBioscience | #17-0862-82 | 1:200 | APC |
| MHCII  (I-A/I-E) | M5/114.15.2 | eBioscience | #47-5321-82 | 1:400 | APC-Cy7 |
| PD-L1 | MIH5 | eBioscience | #46-5982-83 | 1:200 | PerCP-eFluor710 |

Supplementary Table 2. List of antibodies used in the staining – panel 2.

| **Antibody target** | **Clone** | **Source** | **Catalog no.** | **Dilution** | **Fluorochrome** |
| --- | --- | --- | --- | --- | --- |
| CD45.2 | 104 | eBioscience | #56-0454-82 | 1:200 | AlexaFluor700 |
| CD3e | 145-2C11 | Tonbo Biosciences | 65-0031 | 1:200 | PerCP-Cyanine5.5 |
| CD8a | 53-6.7 | eBioscience | 48-0081 | 1:200 | eFluor450 |
| CD4 | GK1.5 | Becton Dickinson | 564667 | 1:200 | BUV 496 |
| CD69 | H1.2F3 | eBioscience | 25-069 | 1:200 | PE-Cy 7 |
| CD25 |  | eBioscience | 53-0251 | 1:200 | AlexaFluor488 |
| CD44 | IM7 | BioLegend | 103008 | 1:200 | PE |
| PD-1 | 29F.1A12 | BioLegend | 135220 | 1:200 | BV 605 |

Supplementary Table 3. Comparison of PCM patients’ characteristics with STING-negative or STING-positive IHC staining of bone marrow samples.

|  | STING-negative (n=30) | STING-positive (n=27) | p-value |
| --- | --- | --- | --- |
| Sex  female  male | 15 (26%)  15 (26%) | 17 (30%)  10 (18%) | 0.042 |
| BM plasmocytes [%] | 62.5 (±25) | 65 (±24.1) | 0.28 |
| M-spike isotype  IgG  non-IgG | 22 (39%)  8 (14%) | 16 (28%)  11 (19%) | 0.26 |
| M-spike [g/dl] | 3.95 (0-9) | 3.1 (0-9.7) | 0.27 |
| FLC ratio >100  Yes  No | 15 (35%)  10 (23%) | 8 (19%)  10 (23%) | 0.31 |
| β2-microglobulin [mg/l] | 5.84 (±3.66) | 4.71 (±2.75) | 0.22 |
| Calcium [mmol/l] | 2.52 (±0.49) | 2.45 (±0.24) | 0.48 |
| Creatinine [mg/dl] | 1.2 (±1.01) | 1.01 (±0,69) | 0.39 |
| Hemoglobin [g/dl] | 10.0 (6.8-14.5) | 10.3 (6.5-13.1) | 0.56 |
| Osteolytic lesions  present  absent | 23 (40%)  7 (12%) | 18 (32%)  9 (16%) | 0.41 |
| Treatment regimen  VTD  CTD | 9 (16%)  21 (37%) | 10 (18%)  17 (30%) | 0.57 |
| Progression after 1st line treatment  Yes  No | 25 (44%)  5 (9%) | 18 (31%)  9 (16%) | 0.14 |
| Death  Yes  No | 12 (21%)  18 (31%) | 10 (18%)  17 (30%) | 0.82 |

Data are shown as number (percentage), mean (standard deviation) or median (interquartile range). Abbreviations: BM – bone marrow; FLC – free light chain; VTD – bortezomib, thalidomide, dexamethasone; CTD – cyclophosphamide, thalidomide, dexamethasone.


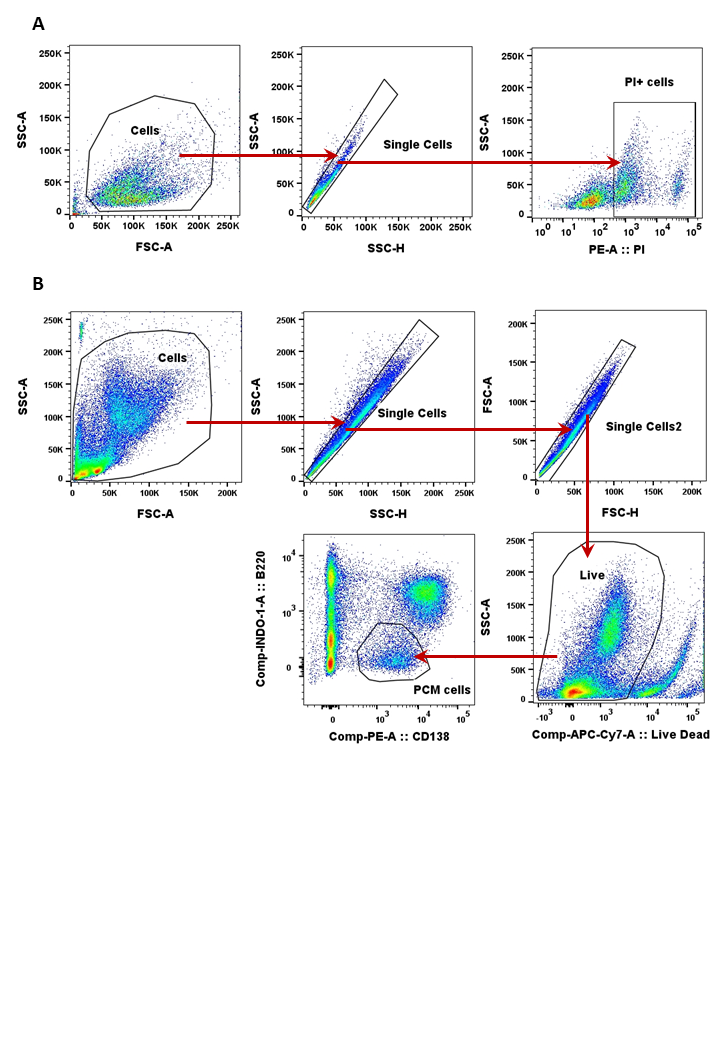


Supplementary Fig. 1. Gating strategy used for identification of PCM cells in the spleen.


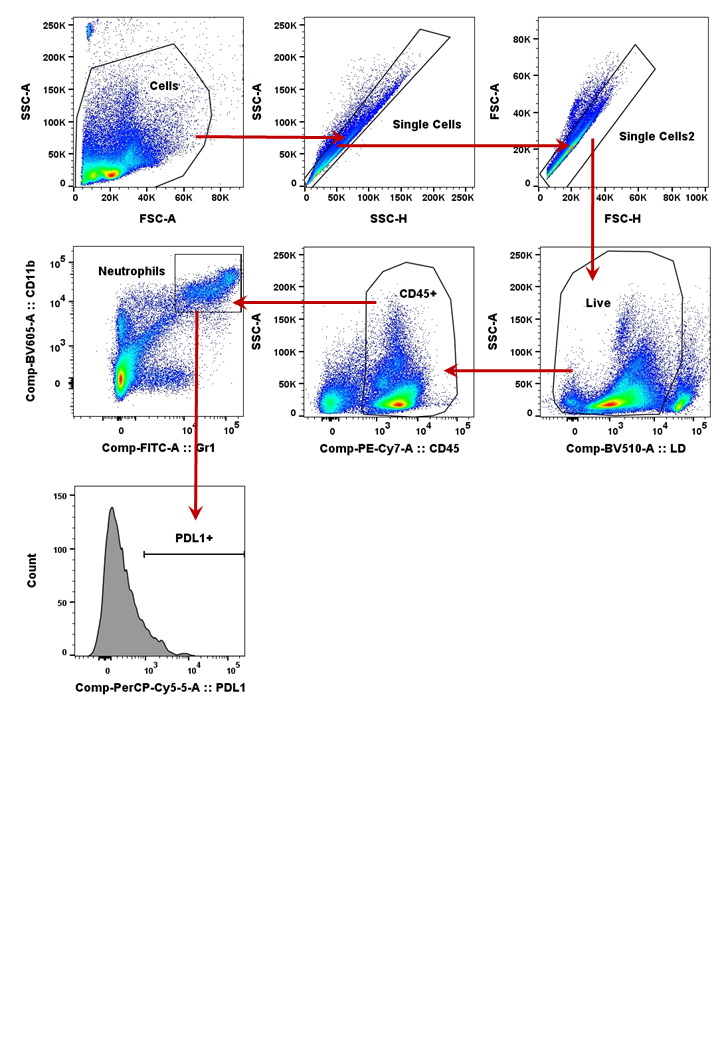
Supplementary Fig. 2. Gating strategy used for identification of splenic neutrophils (live CD45^+^CD11b^+^Gr-1^+^ cells).


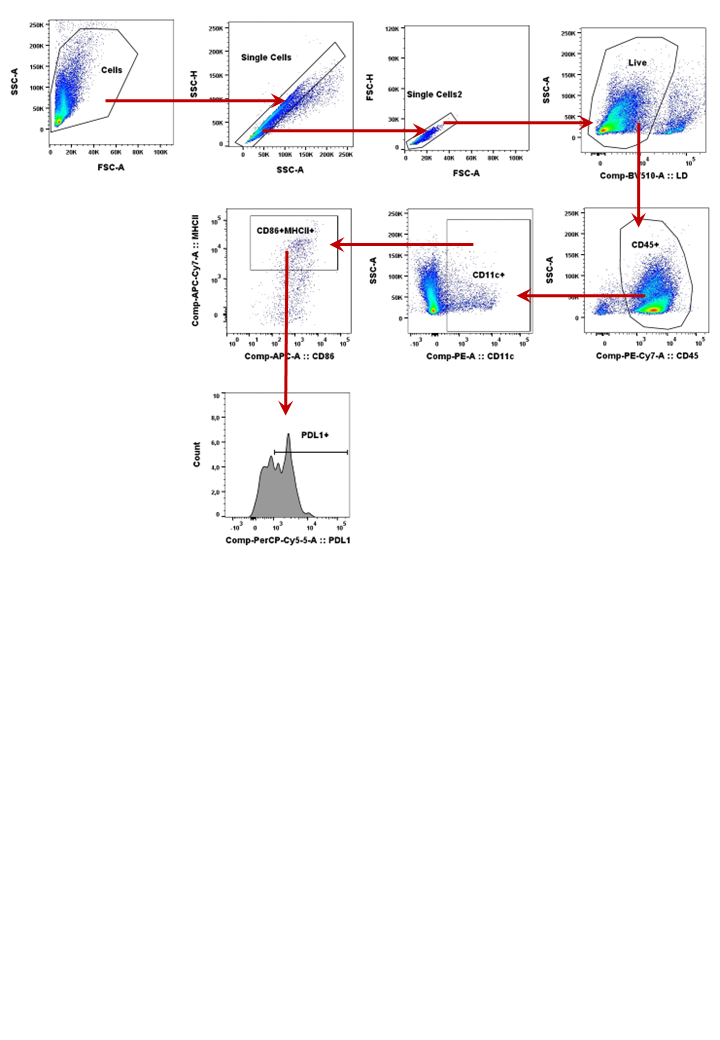
Supplementary Fig. 3. Gating strategy used for identification of activated splenic dendritic cells (live CD45^+^CD11c^+^CD86^+^MHCII^hi^ cells).


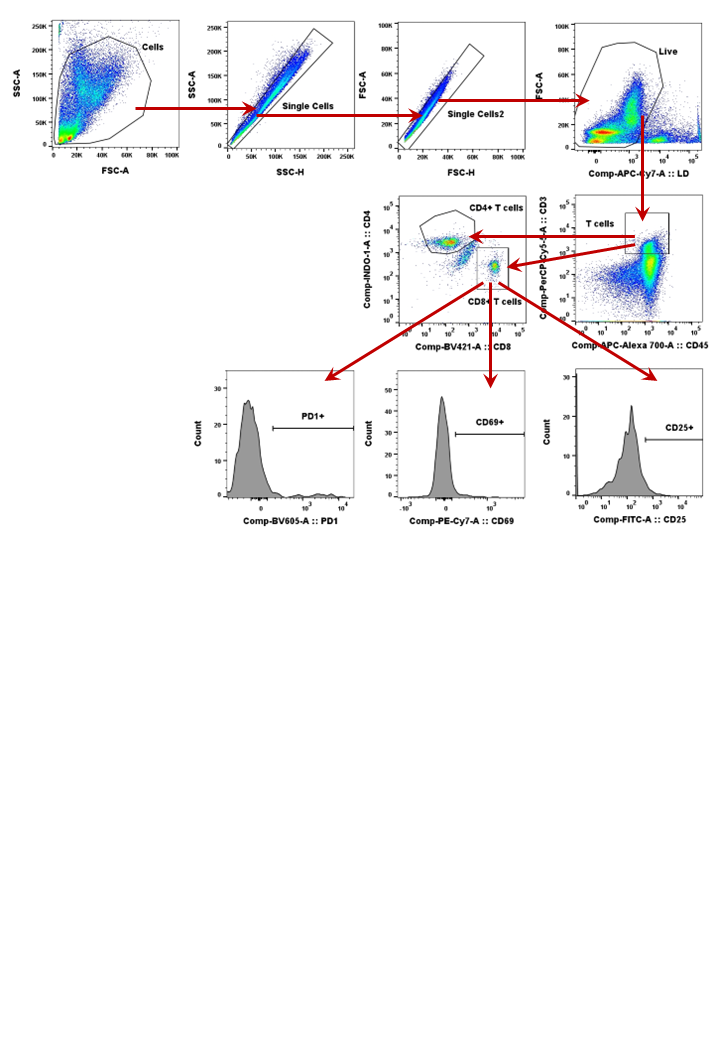
Supplementary Fig. 4. Gating strategy used for identification of splenic CD4^+^ and CD8^+^T cells (live CD45^+^CD3^+^CD8^+^ cells and live CD45^+^CD3^+^CD4^+^ cells, respectively). PD-1, CD69 and CD25 served as T cells activation markers.


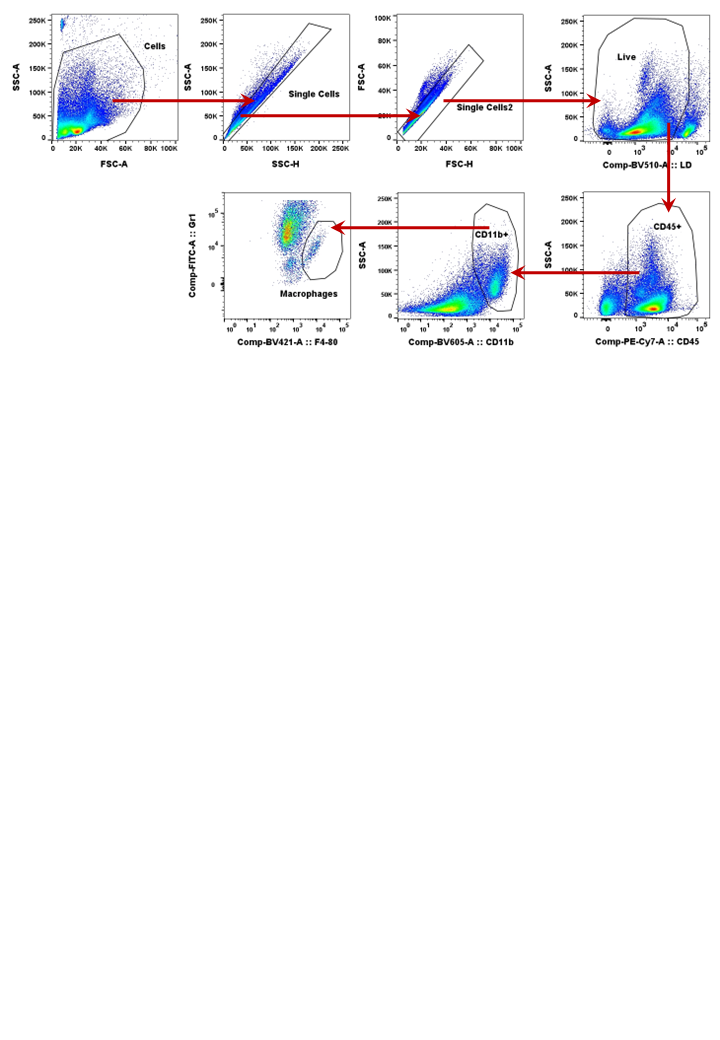
Supplementary Fig. 5. Gating strategy used for identification of activated macrophages (live CD45^+^CD11b^+^Gr-1^-/low^F4/80^+^ cells).


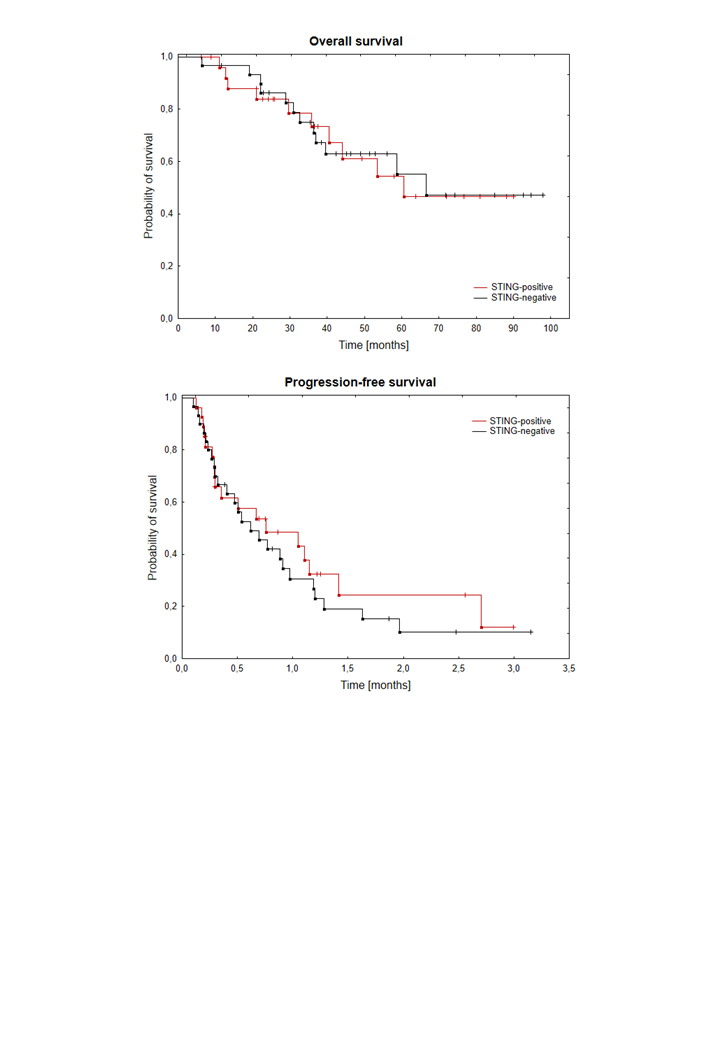
Supplementary Fig. 6. STING expression in PCM cells does not affect overall survival (upper graph) nor progression-free survival (lower graph) in PCM patients. For statistical analysis of clinical parameters of PCM patients, STING samples were divided into two groups: STING-negative (with no IHC reaction) and STING-positive (weak or strong IHC reaction). Survival function with 95% confidence intervals was estimated using the Kaplan-Meyer method.


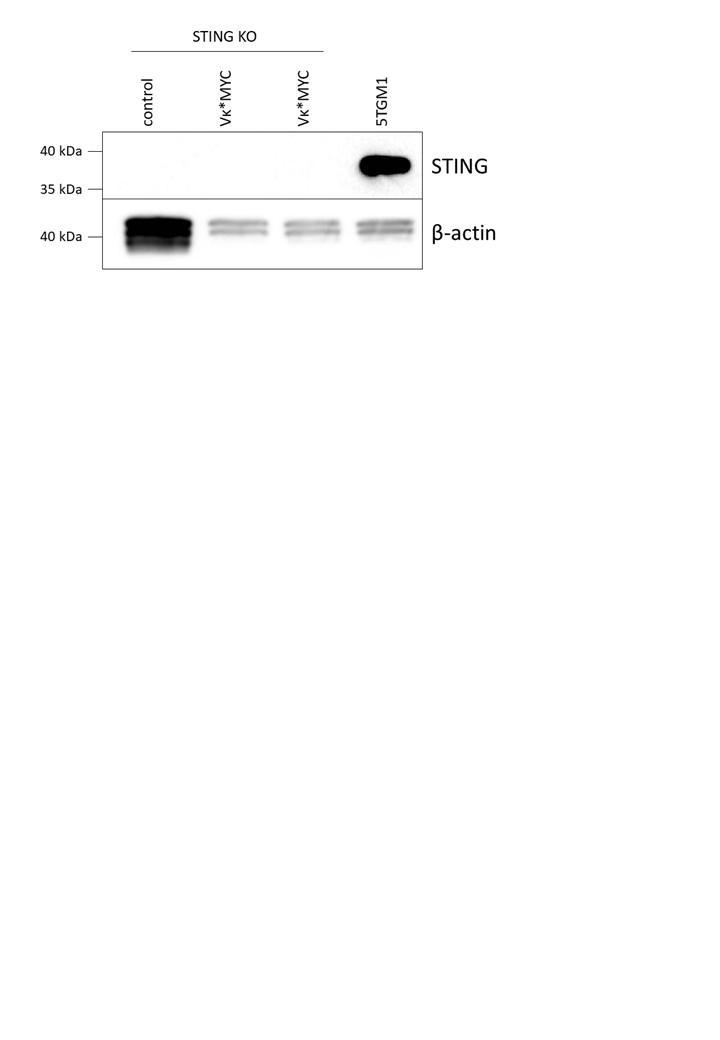


Supplementary Fig. 7. Vĸ*MYC cells do not express STING. Two STING KO mice were transplanted with 0.75x10^6^ Vĸ*MYC cells. After 5 weeks spleens were harvested, lysates were prepared and analyzed by immunoblotting. Lysates of the spleen of a control STING KO mouse and 5TGM1 cells were used as controls. β-actin was used as loading control.


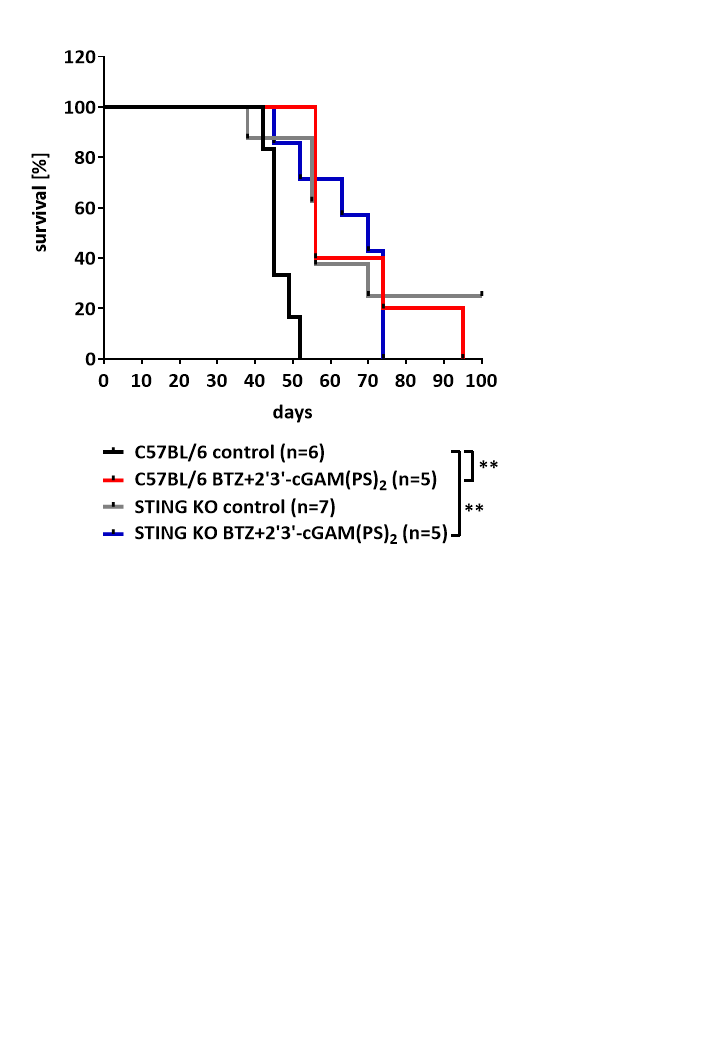


Supplementary Fig. 8. Kaplan-Meier survival curves of mice from experiment described in Figure 2D. **p<0.01, log-rank test.


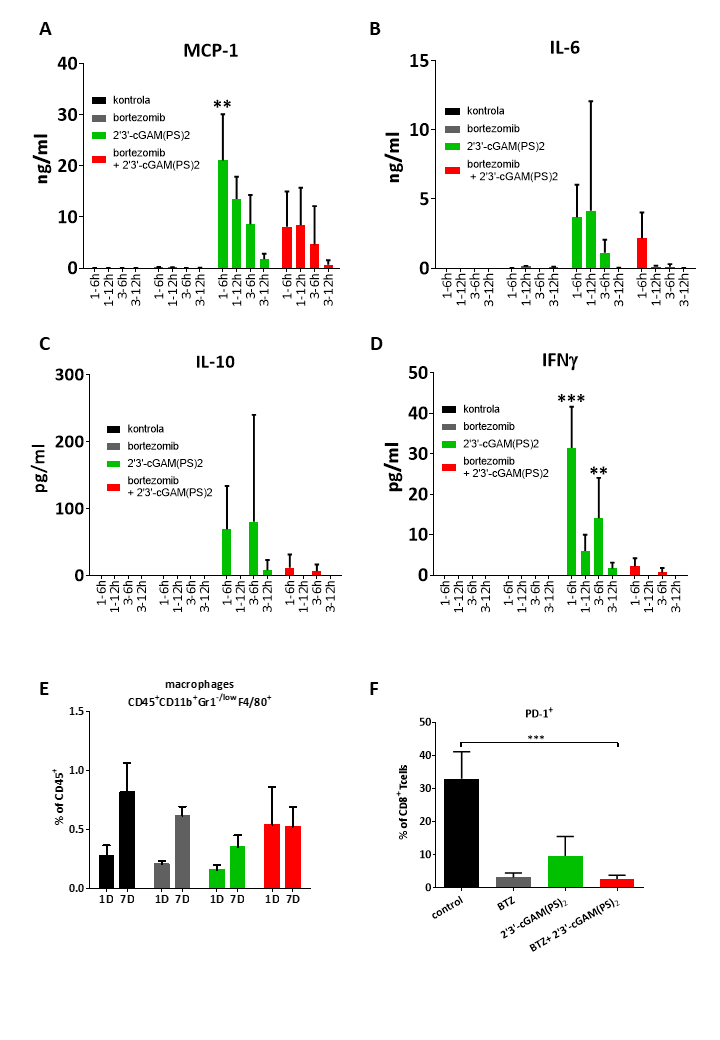
Supplementary Fig. 9. C57BL/6 mice were transplanted with 0,75x106 Vĸ*MYC cells and treated like in Figure 3. Experimental groups consisted of 3 mice. (A-D) Cytokines concentration in serum samples collected 6 or 12 hours after first (1) or third (3) administration of 2’3’cGAM(PS)_2_ was quantified by flow cytometry. Data show means ± SD. **p<0.01, ***p<0.001; one-way ANOVA followed by Dunnett’s post-hoc test versus combination treated group. (E-F) Mouse spleens were harvested 1 or 7 days posttreatment (1D and 7D, respectively). Cells were immuno-phenotyped for macrophages (E) and PD-1^+^CD8^+^ T cells (F). Graph depicts the mean ± SD. ***p<0.001; two-way ANOVA followed by Bonferroni’s multiple comparisons test versus combination treated group.
